# In Situ Programming of CAR T Cells

**DOI:** 10.1146/annurev-bioeng-070620-033348

**Published:** 2021-04-16

**Authors:** Neha N. Parayath, Matthias T. Stephan

**Affiliations:** 1 Clinical Research Division, Fred Hutchinson Cancer Research Center, Seattle, Washington 98109, USA; 2 Department of Bioengineering and Molecular Engineering & Sciences Institute, University of Washington, Seattle, Washington 98195, USA

**Keywords:** CAR T cell therapy, nanoparticle, gene therapy, off-the-shelf T cell therapy

## Abstract

Gene therapy makes it possible to engineer chimeric antigen receptors (CARs) to create T cells that target specific diseases. However, current approaches require elaborate and expensive protocols to manufacture engineered T cells ex vivo, putting this therapy beyond the reach of many patients who might benefit. A solution could be to program T cells in vivo. Here, we evaluate the clinical need for in situ CAR T cell programming, compare competing technologies, review current progress, and provide a perspective on the long-term impact of this emerging and rapidly flourishing biotechnology field.

## INTRODUCTION

Adoptive T cell therapies are a powerful modality in which a patient’s own T cells or those of a donor are harvested, genetically enhanced ex vivo, and then reintroduced into the patient to fight off specific cancers or infectious agents. The efficacy of this approach is supported by numerous clinical trials showing impressive outcomes (1–4). Two T cell products, each expressing a different cancer-targeting chimeric antigen receptor (CAR), are already being used in the United States. They are Kymriah^®^ (tisagenlecleucel) from Novartis AG, which is used to treat children with acute lymphoblastic leukemia, and Yescarta^®^ (axicabtagene ciloleucel) from Gilead Sciences, which is used to treat adults with large B cell lymphoma. However, an annual course of treatment comes at a steep price: $475,000 for Kymriah and $373,000 for Yescarta. Consequently, many patients cannot afford these treatments. The high costs and complexity involved in manufacturing a bespoke T cell product for each patient limit the wider use of adoptive T cell therapies and their competitiveness with frontline therapeutic options, such as small molecule drugs or monoclonal antibodies, which can be produced in bulk quantities as conventional pharmaceuticals can (5). Most CAR T cells and T cell receptor (TCR)-engineered T cells are currently made by a cumbersome process involving the following: (*a*) leukapheresis to extract T cells from a patient who is connected by two intravenous lines to an apheresis machine for several hours (6) (this is uncomfortable for the patient, is expensive, and ultimately may be rate limiting for large-scale adoption of autologous T cell therapy); (*b*) activation and transduction of T cells; (*c*) expansion of transduced T cells in cytokine-supplemented tissue culture medium; (*d*) washing and concentrating the T cells prior to administration (for T cell products made at central facilities and transported to distant treatment centers, the cells must be cryopreserved); and (*e*) quality control release assays, which are necessary for each batch of CAR T cell product. The entire production process has to be conducted according to strict, environmentally controlled good manufacturing practices (GMP) at a central, accredited location, which may be far from the medical center where the patient is located. Because each bespoke CAR T cell product is made from material (T cells) obtained from the patient to be treated, there are no economies of scale (7).

## CLINICAL NEED FOR DIRECT IN SITU PROGRAMMING

### Rapid Antitumor Efficacy

So far, medical science has no procedure that can rapidly educate the immune system to eliminate cancer. For example, vaccines may require months to elicit responses, during which the disease can become fatal (8–10). Likewise, adoptive cell transfer requires a considerable amount of time to expand transfected T cells. Recombinant antibodies can bring about immediate antitumor responses, but as a passive form of treatment their benefits require repeated or even lifelong administration because they are rapidly cleared from the circulation (11, 12). Several groups of investigators have developed bispecific antibodies, including bispecific T cell engagers (BiTEs), dual-affinity retargeting proteins, and diabodies, to redirect resident T cells toward tumor cells (13–15). Among these, blinatumomab (a CD19-specific BiTE) has shown encouraging results for hematological malignancies (16). However, BiTEs must be administered as a continuous infusion and can produce systemic toxicities (16). Furthermore, BiTEs do not undergo active biodistribution or self-amplification following infusion. By contrast, the in situ programming systems reviewed below can generate tumor-specific T cells de novo that actively localize to the target, increase in number, and serially destroy cancer cells. Furthermore, whereas vaccines rely on existing repertoires of T cells, which often express T cell receptors that have low affinity to self/tumor antigens, direct in situ programming can not only equip lymphocytes with engineered receptors that possess high affinity for the tumor target but also provide costimulatory signals to elicit robust T cell expansion.

### Practicality

Given the manifold challenges that cancer already poses to the health care system, it is clearly not possible to provide personalized T cell therapy to every cancer patient who might benefit (17). However, there is a promising alternative—an off-the-shelf injectable reagent designed to equip circulating T cells in situ with a CAR construct that targets a disease-specific antigen expressed by a tumor cell or a pathogen. Such reagents, known as in situ CAR T cell programming nanoparticles, could be readily fabricated in a stable form on a large scale. They would be easy to distribute, would be inexpensive to administer, and could be delivered to sizable patient populations in out-patient settings. Furthermore, preconditioning the recipient is avoided, in sharp contrast to most conventional adoptive CAR T cell therapies, whereby patients must undergo harsh chemotherapy preconditioning to remove endogenous lymphocytes, which otherwise act as cytokine sinks (18). In the case of in situ CAR T cell programming approaches, such preparative procedures are not necessary because existing populations of circulating T cells are directly programmed by the transgene-bearing nanoparticles. During this reprogramming, T cells never exit their physiological environment and are not exposed to supraphysiological levels of cytokines as happens during ex vivo propagation. Instead of undergoing rounds of in vitro expansion—a process that can functionally exhaust cells before they are reintroduced (19)—CAR T cells programmed in situ are immediately poised to eliminate cancer cells. This means that clinicians could initiate anticancer immune responses immediately after diagnosis, not only reducing technical requirements and costs (attributes becoming more and more important for modern immunotherapies) but also potentially making a critical difference for patients whose disease is advancing rapidly.

### Treatment Resistance

One limitation of CAR-based T cell therapies is the phenotypic diversity of tumors, which means some cancer cells are not recognized by the targeting receptors and so form immune escape variants (20–22). Programming lymphocytes with CARs that recognize different tumor antigens could address this problem, but generating a spectrum of T cell variants is not practical using ex vivo procedures. On the other hand, injectable CAR T cell programming reagents offer a simple way to rapidly generate immunity against any targetable tumor antigen: Oncologists could fine-tune multiple T cell specificities so they match the antigenic fingerprint of each patient’s tumor.

## VIRAL PLATFORMS

**Figure 1:**
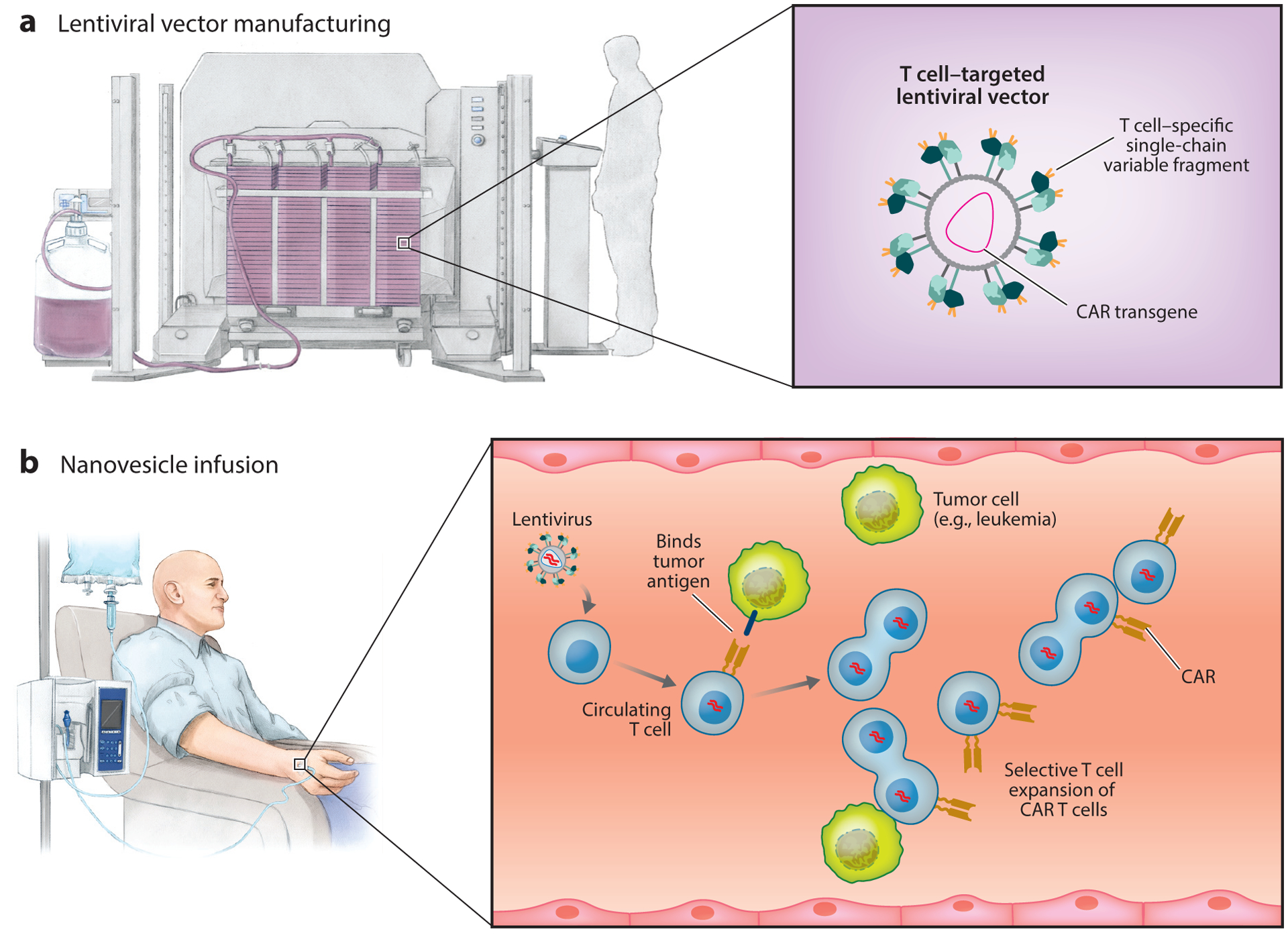
Viral vectors (T cell targeting/DNA integrating). (*a*) Production of chimeric antigen receptor (CAR)–encoding lentiviral vector. To target lymphocytes, vectors are pseudotyped with engineered glycoproteins that recognize lymphocyte surface markers as entry receptors. (*b*) Systemic infusion of lentivirus results in the transduction of a small number of circulating T cells, which subsequently undergo clonal proliferation and differentiation into effector cells following antigen encounters.

To generate CAR T cells ex vivo, researchers find lentiviral vectors particularly appealing due to their ability to stably integrate relatively large DNA segments into the host cell genome and to efficiently transduce not only dividing cells but also nondividing cells (Figure 1) (23). However, infusing patients directly with lentiviral vectors to deliver CAR transgenes into circulating T cells is challenging. Lack of selectivity, innate immune responses against the viral particle (24), inactivation by human serum complement (25), and the risk of uncontrollable vector-based insertional mutagenesis (26) all pose major hurdles to the clinical translation of this approach. The key to reducing the risk of insertional mutagenesis and malignancy is to avoid in situ gene transfer into off-target hematopoietic stem cells or progenitor cells (since these are long-lived and more prone to oncogenesis) and restrict the gene delivery specifically to T cells. Exclusive cell targeting cannot be accomplished using vesicular stomatitis virus G (VSV-G) pseudotyped lentivirus vectors that are routinely used for the ex vivo transfer of CARs into human T cells for clinical applications. This is because the VSV-G protein has a broad tropism, mediated through cell-surface heparan sulfate, which is found on all cell types (27).

To restrict the tropism of CAR-encoding lentiviral vector to T cells, Buchholz and colleagues (28, 29) pseudotyped the vector with modified envelope proteins of Nipah virus or measles virus. Cell-type specificity was achieved by fusing these envelope proteins to a CD8- or CD4-specific single-chain variable fragment. They showed that a single intravenous infusion of 2.5 × 10^11^ CD8-targeted lentiviral particles encoding a CD19-specific CAR (targeting B cells) substantially reduced the tumor burden in B cell lymphoma-bearing immunodeficient NOD scid gamma (NSG) mice that had been engrafted with human T cells prior to virus infusion (30). Tumor regression correlated with ~10% of in situ reprogrammed host CD8 T cells stably expressing the CD19-specific CAR, while CAR expression in CD4 T cells was undetectable. But, as demonstrated by the same group in an earlier report (31), these high percentages of CD8^+^ CAR T cells generated in vivo were not the result of efficient in situ gene transfer; in fact, the size of the starting populations of successfully reprogrammed CD8^+^ CAR T cells following CD8-lentivirus infusion was usually very small (less than 0.5%). Instead, as one would hope, the CAR-transduced cells subsequently underwent selective clonal proliferation and differentiation into effector T cells following antigen encounters. To confirm the utility of this platform in a more physiological test system, Buchholz and colleagues (31) infused CD34^+^ humanized NSG mice, which develop multilineage human immune cells, with CD8 lentiviral vectors that express the CD19-specific CAR. In this experiment, 7 of the 10 treated animals had reduced numbers of circulating B cells, which correlated with increased levels of inflammatory blood cytokines. Although biodistribution, immunogenicity, and off-target gene transfer into different tissues (e.g., heart, lungs, liver) of the infused viral vectors were not assessed, the study established an important proof of concept: that injection of T cell–targeting lentivirus can deliver CAR transgenes into enough host T cells to induce a therapeutic response.

### Advantages

The success of established CAR T cell therapy has been heavily dependent on ex vivo lentivirus-mediated gene transfer. Thus, the development of lentiviral vectors as an injectable reagent for in situ reprogramming of T cells was an obvious next step for the reasons outlined below.

#### Established manufacturing.

Lentiviruses are becoming the gene-transfer vehicle of choice for use in the treatment of a variety of genetic and acquired human diseases (32). Lentiviral vectors are advantageous for generating CAR T cells ex vivo owing to the reasons already noted above, that is, their ability to stably integrate large DNA inserts and to efficiently transduce both dividing and nondividing cells (33). Growing interest in the use of lentiviruses has created a strong demand for large volumes of concentrated vector for use in clinical trials. As a result, scalable production platforms as well as characterization tests are now in place to manufacture clinical-grade lentiviral vectors (34–36).

#### A structure preadapted for efficient gene transfer.

Lentiviral vectors are complex bioproducts with an ordered architecture that would be challenging to engineer using synthetic material. For example, the surface of the virus particle displays all the targeting ligands in the desired orientation and density to achieve efficient cell targeting. Furthermore, after binding to the receptor on the target cell, lentiviruses are inherently efficient at becoming internalized into the cytoplasm through a variety of endocytic pathways (37). These steps, which lentiviral delivery has already overcome, are all critical hurdles during the engineering of synthetic nucleic acid delivery systems.

### Potential Shortcomings

Despite the advantages outlined above, systemic delivery of lentiviruses presents major drawbacks.

#### Safety.

Producing a GMP-grade lentiviral vector preparation for human use requires rigorous testing of the final product to establish its purity, potency, and safety (26, 38). In contrast to analyzing most other pharmaceuticals, analyzing a biologically active viral vector creates particular challenges. These vectors tend to favor integration at genome sites near active genes, which carries the risk of disrupting the transcriptional regulation of neighboring regions in some genomic contexts. Both preclinical (39) and clinical (40, 41) data support a precautionary approach in patients, despite no reports of T cell transformation associated with human immunodeficiency virus transgene expression in the large numbers of patients treated with that system (42). Moreover, the produced vector batches must be titrated and tested for replication-competent lentivirus, in a process that is labor-intensive and increases the final cost of the therapy. Due to the theoretical possibility of secondary malignancies, health authorities in the United States and Europe require long-term follow-up for studies of cell and gene therapies that use viral vectors. The requirements for follow-up vary depending on the country; in the United States, monitoring of patients at least once a year for 15 years after receiving gene therapy is recommended (43).

#### Cost.

Modified viruses are exacting to make in the clinical setting, and there are now long wait lists for these vectors. A major drawback to their large-scale manufacturing is the presence of defective virions among fully biologically active virions recovered from the culture medium (44). This tainting is a dynamic process involving both production and inactivation rates, and it ultimately yields some 3 to 10 functional virus particles per producer cell (45). In contrast, during natural lentiviral infection, the number of virions is close to 10^3^ per cell (46). In one report, Buchholz and colleagues (47) described a 100-fold lower viral titer of CD8-targeting lentiviral vectors compared with VSV-G pseudotyped lentivirus. Using the current platforms of production and purification necessitates generating an enormous volume of viral supernatant to ensure sufficient quantities of particles to treat a patient with CAR-programming lentivirus.

#### Immunogenicity.

Lentiviral particles are assembled in a complex process. In the final step, the outer envelope of the virus is formed from the plasma membrane of the packaging cell as the virion exits the cell by budding. Thus, the lentiviral envelope, in addition to having viral antigens, contains an array of host-membrane proteins that may act as immune triggers upon recognition and phagocytosis by professional antigen-presenting cells (48). This may limit vector stability in patient serum, prevent redosing, and exclude from treatment patients who have preexisting antibodies against the lentiviral vector. To address this issue, the Naldini group (49) applied gene editing to packaging cells to generate alloantigen-free lentiviral vectors. The ability to administer booster doses of CAR-programming reagents is likely to be crucial, especially in cancer patients with rapidly progressive disease. Redosing would allow physicians to either reinforce the immune attacks or alter the immune response as the disease evolves.

## NONVIRAL PLATFORMS

### T Cell–Targeted Nanoparticles

Conceptually, nanoparticles are ideal off-the-shelf reagents that are able to quickly reprogram T cells to recognize and destroy tumors without the need for laboratory manipulations postsyn-thesis. Their morphology and composition can be customized, and instruments for the large-scale production of nanomaterial have been developed. Originally, nanotechnology-based clinical research focused on particles that selectively accumulate chemotherapeutics, small interfering RNA (siRNA), or imaging agents at tumor sites, in an effort to minimize off-target toxicities (50–52). With the rise of immunobioengineering as a new discipline, scientists are now developing a variety of injectable nanosystems as a new approach to deliver therapeutic drug doses to circulating T cells or to track their movement (53). The Saltzman group at Yale (54), for instance, described in 2007 a T cell–binding reagent built from a hydrophobic dendrimer core with radiating polyethylene glycol chains terminated with functional amines, which were coupled to streptavidin and surface-functionalized with biotinylated antibodies that recognize the T cell CD3 complex. Similar T cell–targeted polymer- or liposome-based nanoparticles have been engineered by other groups specifically to deliver siRNA (55), small molecules (56), or immune-modulatory drugs to either CD4^+^ or CD8^+^ lymphocytes (57).

**Figure 2:**
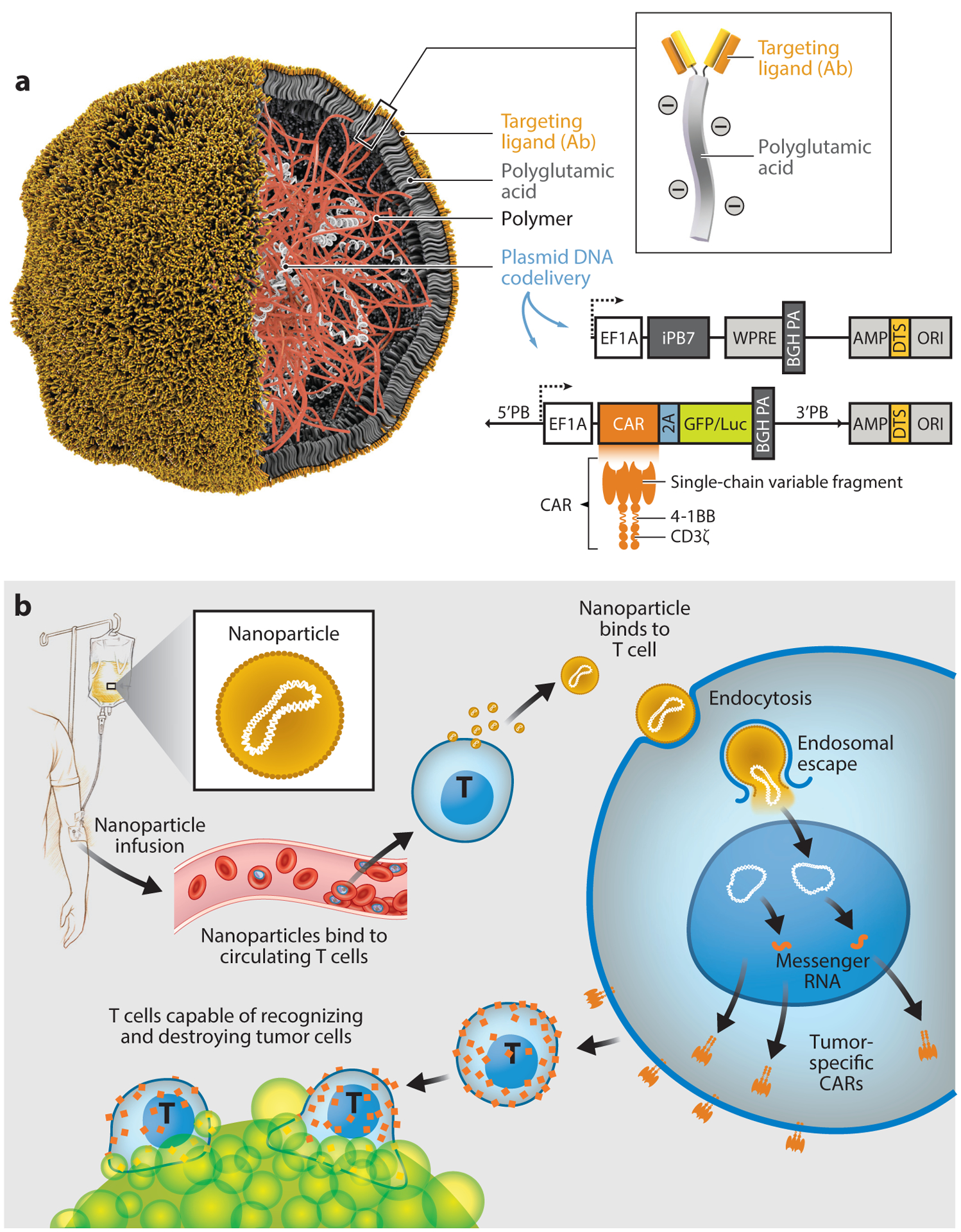
Nonviral vectors (T cell targeting/DNA integrating). (*a*) Schematic of the T cell–targeted DNA nanocarrier. Also depicted are the two plasmids that were encapsulated into the nanoparticles; these encode the CAR and the hyperactive iPB7 transposase. (*b*) Schematic illustrating how to reprogram T cells in situ to express tumor-specific CARs using genes carried by polymeric nanoparticles. These particles are coated with ligands that target them to cytotoxic T cells, so once they are infused into the patient’s circulation they can transfer the genes they carry into the lymphocytes and program them to express the tumor-targeting CARs on their surfaces. Abbreviations: AMP, ampicillin resistance gene; BGHPA, bovine growth hormone polyadenylation signal; CAR, chimeric antigen receptor; DTS, DNA-targeting sequences; EF1A, eukaryotic translation elongation factor 1 alpha 1; iPB7, hyperactive piggyBac transposase; ORI, origin of replication; WPRE, woodchuck hepatitis virus posttranscriptional regulatory element.

Our group adapted these platforms to design injectable DNA nanocarriers that choreograph robust CAR production in host T cells (Figure 2). To create a reagent that can genetically modify primary T lymphocytes (which are notoriously refractory to nonviral transfection methods) simply by contact, we bioengineered polymeric nanocarriers with four functional components:

1. Surface-anchored targeting ligands that selectively bind the nanocarriers to T cells and initiate rapid receptor-induced endocytosis to internalize them: In our studies, we used anti-CD3ε F(ab′)2 fragments as well as full-length anti-CD8 antibodies with deactivated constant regions (Fc).
2. A negatively charged coating that shields the nanocarriers to minimize off-target binding by reducing the net surface charge of the nanocarriers: We used polyglutamic acid to accomplish this in our experiments.
3. A carrier matrix that condenses and protects the nucleic acids from enzymatic degradation while they are in the endosome but releases them once the particles are transported into the cytoplasm, thereby enabling transient transcription of the encoded protein: Our group tested a panel of cationic materials, including hyperbranched star polymers (58), polyethylene glycol–grafted polyethylenimine (59), and mesoporous silica nanoparticles (60), and selected a biodegradable poly(β-amino ester) polymer formulation because of its superior transfection efficacy and low biomaterial-mediated cytotoxicity in primary T cells. The lack of poly(β-amino ester) toxicity is a result of its high biodegradability, as it has a half-life between 1 and 7 h in aqueous conditions (61). This time frame is ideal for gene therapy, as the polymer condenses and effectively protects nucleic acids against degradation while in the endosome but releases them once the nanoparticles come in contact with the cytoplasm, thus enabling expression of the encoded protein.
4. Nucleic acids that are encapsulated within the carrier and produce expression of a disease-specific CAR: To achieve persistent CAR expression in actively dividing T cells, we loaded each nanoparticle with two DNA plasmids: one encoding a mouse version of a CAR that confers specificity for CD19 [the target expressed by leukemia cells and normal B cells (62)] and a second encoding a transposase gene to enable integration of the CAR DNA into the host genome (63).

We compared this nanoparticle system with CAR T cells generated ex vivo (using a protocol that mimics clinical trials) in mice with established CD19^+^ leukemia and found the therapeutic efficacy to be similar (64). A significant proportion of the nanoparticles was taken up by nonreceptor-mediated mechanisms into phagocytic cells in the liver and spleen. However, while the fraction of phagocytes expressing CARs rapidly declined over time, the number of CAR T cells continued to rise. This expansion was remarkable considering the nanoparticles were administered without standard preconditioning chemotherapy to deplete endogenous lymphocytes. Thus, the proliferative stimuli from high levels of CD19 on numerous leukemia cells might compensate for the lower gene-transfer efficiency of nonviral systems. As the study was performed in immunocompetent mice, we could also explore the immune-toxicity potential of the nanoparticles. Although we found modest increases in the levels of the inflammatory cytokines interleukin-12 and interleukin-6 in circulation, no treatment-related lesions were detected by histopathology, and the cell counts and blood chemistry suggested no systemic toxicity.

**Figure 3:**
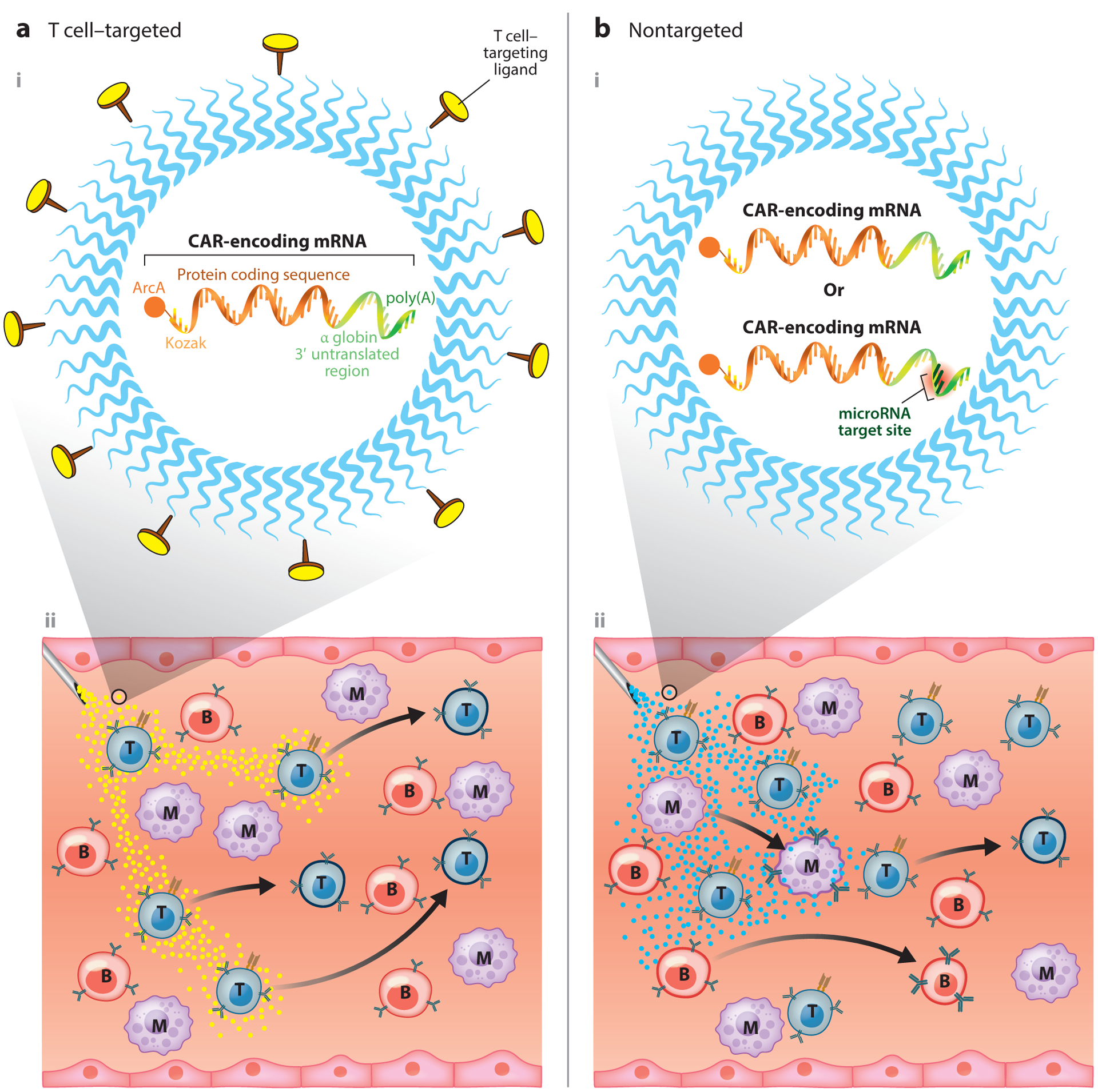
Nonviral vectors (nonintegrating). (*a*) Lymphocyte-targeted nanoparticles. (*i*) Shown is a nanocarrier loaded with in vitro–transcribed mRNA for the transient in situ reprogramming of T cells with disease-specific CARs. Surface-anchored targeting ligands selectively bind the nanoparticles to T cells and initiate rapid receptor-induced endocytosis to internalize them. (*ii*) Following infusion, these nanoreagents (*yellow dots*) quickly and specifically program antigen-recognizing capabilities into circulating T cells. In contrast to DNA nanocarriers, synthetic mRNA molecules are directly translated into therapeutic target proteins without the need to enter the nucleus, ensuring high transfection rates and rapid therapeutic effects. (*b*, *i*) Nontargeted nanoparticles. (*ii*) Due to the lack of a targeting ligand on their surface, these particles (*cyan dots*) nonspecifically transfect circulating blood cells, including T cells, B cells, monocytes, eosinophils, neutrophils, and granulocytes. Transient CAR expression reprograms these cells into new functions and phenotypes. Specific microRNA targeting sequences could be included in the synthetic mRNA construct to ensure translation is limited to therapeutically desirable target cells. Abbreviations: ArcA, antireverse cap analog; B, B cell; CAR, chimeric antigen receptor; M, monocyte; mRNA, messenger RNA; T, T cell.

In a follow-up study (65), we explored the use of in vitro–transcribed (IVT) messenger RNA (mRNA) to quickly and specifically program antigen-recognizing capabilities into circulating T cells as a strategy to treat cancer and infectious disease (Figure 3a). IVT mRNA has recently come into focus as a potential new drug class to deliver genetic information (66, 67). Such synthetic mRNA medicines structurally resemble natural mRNA and can be engineered to transiently express proteins. They are easily developed, inexpensive, and scalable for manufacturing purposes. Advances in addressing the inherent challenges of this drug class, particularly related to controlling the translational efficacy and immunogenicity of the IVT mRNA (68–71), are availing the technology to a broad range of potential applications. The clinical development of mRNA-based therapeutics has also been accelerated at university spin-off companies (e.g., Argos Therapeutics, BioNTech, CureVac, eTheRNA, Ethris, Factor Bioscience, Moderna Therapeutics, and Onkaido), which are supported by considerable venture capital inflows to develop mRNA-based cancer immunotherapies and infectious disease vaccines. In contrast to DNA nanocarriers, synthetic mRNA molecules are directly translated into therapeutic target proteins without the need to enter the nucleus, ensuring high transfection rates and rapid therapeutic effects. Their trim size allows a high copy number per nanoparticle. Also, uncontrolled insertional mutation and promoter dependency are avoided because the delivered mRNA exerts its function in the cytoplasm. Using clinically relevant preclinical models of leukemia, prostate cancer, and hepatitis-induced hepatocellular carcinoma, we demonstrated that repeated infusions of rationally designed mRNA nanocarriers can selectively deliver CAR genes into host T cells and program them in quantities sufficient to bring about disease regression with efficacies similar to those of adoptive methods.

### Advantages

Targeted nanoparticle-based gene modification systems exhibit some key features that could help steer this field toward clinical applications.

#### Customizable platform.

Synthetic nanoparticles are designed in a modular fashion, which facilitates switching out of individual modules and incorporating custom modalities without interference from the biological constraints that viral vectors face. Depending on the desired characteristics, nanoparticles can be fabricated with organic materials such as protein, polymer or lipid, or inorganic compounds (e.g., gold or silica). Also, the therapeutic cargo encoding the CAR can be customized; the cargo can range from IVT mRNA for transient in situ gene expression to constructs that deliver a transgene into the nucleus for persistent gene expression, such as linear, closed-ended DNA (72) or minicircle vectors (73). Similarly, a full range of T cell–binding proteins or synthetic antibody mimetics can be considered during the design of targeted nonviral gene-delivery systems. Examples include designed ankyrin repeat proteins (74), affimers (75), and aptamers, all of which have recently been reported to selectively bind human CD8 T cells (76).

#### Scale-up.

For injectable CAR-programming reagents to be competitive with lentiviral products, it will be vital to create sufficient quantities for clinical use at substantially lower costs than those entailed in producing conventional ex vivo engineered CAR T cells. Several continuous-flow microfluidics platforms designed for scalable manufacturing of nanoparticles under GMP conditions are now available (77, 78). These instruments enable scale-independent synthesis of nanoparticles that can be increased from milligram to gram amounts in a single day (79).

### Potential Shortcomings

While injectable nanoreagents could enable physicians to rapidly program circulating T cells with disease-specific CARs, translation of this nanomedicine into the clinical setting may still be challenging for the reasons outlined below.

#### Need for repeated dosing.

The gene-transfer efficiency of nonviral systems is usually less than that of viral systems (80), which means that patients will likely need repeated dosing to program CAR T cells in quantities that are sufficient to bring about tumor regression. Viral particles possess innate machinery to overcome cellular barriers (cellular uptake, endosomal escape, nuclear entry, and nucleic acid release). Overcoming the same barriers with rationally designed nucleic acid nanoformulations requires great effort and is often challenging. In our hands, in vitro gene transfer into mouse T cells using high-titer lentivirus was around twofold higher compared with the use of T cell–targeted polymeric nanoparticles containing piggyback transposable elements (64). It would be worth testing nonviral delivery of more advanced DNA constructs, such as closed-ended DNA, that consist of an expression cassette flanked by adeno-associated virus inverted terminal repeats to facilitate movement from the cytoplasm to the nucleus to mediate durable high levels of gene expression (72).

#### Multicomponent manufacturing with good manufacturing practices.

In contrast to therapeutics that are already established in the clinic, such as small molecules or antibodies, CAR-programming nanoparticles are multicomponent three-dimensional constructs that require a reproducible manufacturing process to reliably achieve the intended physicochemical characteristics, biological behaviors, and pharmacological profiles. The safety and efficacy of such nanomedicines can be influenced by minor variations in many parameters and need to be carefully monitored, particularly in the context of targeting to unintended sites and potential toxicities. Furthermore, nanomedicines require additional developmental and regulatory considerations compared with conventional medicines. Only a few facilities with the requisite degree of expertise are currently operational in the United States.

## NANOPARTICLES WITHOUT T CELL–TARGETING LIGANDS

Researchers at Moderna Therapeutics are pursuing a nontargeted approach to program CAR T cells by repeated infusion of untargeted mRNA nanoparticles (Figure 3b). The company gave some details of its Immune Nanoparticle program at a Science Day event in May 2019. Moderna Therapeutics presented preclinical data showing it could deliver mRNA to 10–20% of circulating T cells, natural killer (NK) cells, B cells, and various myeloid cells in vivo in animals and ex vivo in human blood (81). The rationale for this sweeping approach to equip all major immune cell types with CARs is predicated on preclinical data from other groups showing that CAR constructs can be activated in non-T cells and trigger antitumor activity in them. Work done by Gill and colleagues (82), for instance, established that CAR-modified macrophages can infiltrate solid tumors and engulf cancer cells. To engineer macrophages from cancer patients with tumor-specific CARs, researchers isolate cells from patient blood and transduce them with a chimeric adenovirus. This vector not only delivers the CAR transgene but also polarizes macrophages toward an immunostimulatory M1-like phenotype. A recent study showed that NK cells also may be genetically engineered with CARs (83). This work has led to the first immunotherapeutic approaches to cure both lymphomas of B cell origin and acute lymphoid leukemia. These approaches involve human NK cells of different origins, such as the NK cell line NK-92, primary cord blood–derived NK cells, and peripheral blood NK cells, which have recently entered phase I/II clinical trials to test their effectiveness (84). To overcome the lack of a T cell–targeting ligand on the surface of their nanoparticles, Moderna Therapeutics is exploiting tissue-specific differences in the expression of endogenous microRNA. Incorporating complementary microRNA target sites into the 3′untranslated region of their IVT mRNA cargo can at least partially suppress protein expression in off-target cells (85).

Another interesting approach to help constrain nanoparticle transfection to T cells in vivo without using targeting ligands was described by Dahlman’s group (86). This team synthesized libraries of chemically diverse lipids and then investigated which lipid nanoparticles manufactured from these libraries can deliver siRNA preferentially to T cells. To directly screen a large number of different liposome formulations in vivo, the researchers DNA-barcoded nanoparticles and loaded them with siRNA against green fluorescent protein. Using mice that constitutively express green fluorescent protein, they were able to evaluate which distinct lipid nanoparticles delivered siRNA to any combination of target cells. Although no exclusive uptake of nanoparticles by T cells was observed in these screens, an adamantane-containing liposome formulation (dubbed 11-A-M) achieved high transfection rates of splenic T lymphocytes. The theory underpinning this approach is that different cell types have different natural trafficking pathways that promote delivery of specific kinds of lipid nanocarriers. Validation of this theory, the mechanism of 11-A-M liposome tropism to T cells, and whether human T cells can be selectively transfected using the same nanoreagent remain to be addressed.

### Advantage

Biotechnology companies developing conventional nanomedicines generally try to avoid surface functionalization of the nanocarriers, as it complicates scale-up manufacturing and quality control and adds cost. Manufacturing T cell–specific targeting ligands in a GMP facility is expensive and time consuming. To avoid immunogenicity, the chosen constructs must be fully humanized. Furthermore, to maximize targeting efficiency, a GMP-compliant coupling strategy must be developed to tether the targeting ligands to the surface of the nanoparticles while ensuring they are displayed in the appropriate orientation to allow the ligand to bind its receptor on T cells. In contrast, simple methods are already established for mass production of untargeted liposomes (87, 88).

### Potential Shortcomings

Intravenous infusion of untargeted nanoparticle formulations could have significant disadvantages over lymphocyte-targeted delivery systems.

#### High off-target gene transfer.

Exploiting receptor-mediated endocytosis is advantageous for achieving targeted gene delivery as it improves both the efficacy and the side-effect profile of CAR-programming nanomedicines. Through the targeting of nanoparticles to specific surface receptor proteins on T cells that are actively internalized, the nanoparticles can be selectively shuttled into endosomes of T cells (89, 90). None of the untargeted gene-delivery strategies described above can achieve selective binding to circulating T cells as the approach is based on random (and often charge-driven) transfection of all cell types encountered by the nanoparticles. Once these nanoparticles have entered a cell, gene expression in non-T cells is reduced by the presence of microRNA response elements or by the selection of lipid compositions that favor trafficking pathways in lymphocytes. Therefore, systemically administered untargeted nanoparticles will likely require a multifold higher dose, compared with T cell–targeted nanoparticles, to achieve equal transfection rates in circulating T cells, as most of the infused dose is internalized by off-target cells.

#### Unpredictable side effects.

The consequences of expression of a CAR protein in non-T cells are unknown at this point. The untargeted nanoreagents described by Moderna Therapeutics and Dahlman’s group (86) deliver transgenes not only into a variety of cell types and tissues, including monocytes, B cells, neutrophils, and eosinophils, but also into nonlymphoid tissues such as the endothelium. Even if the intracellular CD3ζ domain of the CAR construct does not signal in most of these cell types, the surface display of the tumor-antigen-specific single-chain variable fragment recognition domain may alter the migration and tissue tropism of CAR-transfected cells—with uncertain consequences.

## COMPETITIVE LANDSCAPE

Following the success of the patient-derived ex vivo engineered CAR T cell products Kymriah (tisagenlecleucel) and Yescarta (axicabtagene ciloleucel), CAR T cell developers are looking for technologies to provide better scale and lower costs similar to those of biologics. The ability to benefit a large patient population at an affordable price will also be key to move CAR T cell therapy beyond cancer applications. Preclinical studies showing the utility of CAR T cells outside of cancer are ramping up, and developers have taken notice. Autoimmunity is seeing the most activity, followed by infectious disease and transplant settings. Academics are pushing the technology even further, into less obvious indications such as heart failure. The adoption of CAR T cells as a mainstream therapy is still some years away, as the biotechnology industry currently lacks off-the-shelf injectable reagents that do not require complex manufacturing. Instead, the technology is currently limited to allogeneic platforms such as those described below.

### Allogeneic CAR T Cells Manufactured from Healthy Donors

In the hope that allogeneic cells can broaden the reach of CAR T cells to more patients and more types of cancer, most companies involved in clinical-stage autologous T cell therapy are also now developing T cell products from allogeneic healthy donors, rather than from the patient (91,92). The driving force behind the progress has come from advances in orthogonal technologies, such as different forms of gene editing, genetic knockdown, and cell engineering (93). The two main challenges that CAR T cell developers face in transitioning from autologous to allogeneic therapies are graft versus host disease and host versus graft reactions, both of which need to be overcome to create safe and persistent off-the-shelf treatments. In graft versus host disease, TCRs on the transplanted donor CAR T cells recognize the patient as foreign and trigger the donor cells to attack the patient’s healthy tissues (94). The dominant strategy for generating allogeneic cells is therefore to remove the TCR from the donor cells using gene editing or short hairpin RNA. However, companies are discovering that as they eliminate expression of the TCR, they increase the chance that the host immune system will reject the donor cells before they kill the cancer (host versus graft) (92). Consequently, poor persistence of the donor cells severely limits the therapeutic efficacy of the allogeneic T cell product. To mitigate the host versus graft response, it is necessary to remove graft-cell proteins such as CD52, major histocompatibility complex class I molecules, or the major histocompatibility complex component β−2 microglobulin by gene editing or other silencing methods (91).

However, this immunological tailoring of allogeneic CAR T cells through the addition of genome editing and cell purification steps complicates the manufacturing protocol, and this complication not only delays production and increases costs (including costs for gene-editing intellectual property) but also reduces the viability of the lymphocytes and their yield (89). Allogeneic CAR T cells have clear advantages over autologous therapies, particularly in patients with advanced malignancy who have an extensive treatment history and where collection of T cells sufficient for the CAR T cell manufacturing cycle may be difficult. Nonetheless, sourcing T cells from healthy donors instead of patients will not solve the overall challenge of providing large numbers of patients with a mainstream CAR T cell therapy that is affordable, effective, and safe. The promise of creating hundreds of doses of CAR T cells from one batch of healthy donor T cells might be realistic for treatment of pediatric patients with B cell malignancies, where CD19 CAR T cell doses as low as 0.2–5 × 10^6^ transduced viable T cells per kilogram of body weight have been shown to be effective (95, 96). In most clinical applications (e.g., solid tumor, infectious disease) redosing is required to achieve a therapeutic response, and the need for redosing will further increase product demand (97, 98). Since scale-up of allogeneic CAR T cells is limited by the blood volume that can be safely taken from one healthy donor, allogeneic technologies might be made more cost effective than conventional autologous T cell manufacturing by supraphysiologically expanding the final allogeneic CAR T cell product ex vivo before administration. This, however, would likely functionally exhaust the T cells and further reduce their ability to expand, persist, and kills tumor cells in patients (99).

### Allogeneic CAR T Cells Manufactured from Induced Pluripotent Stem Cell Lines

Human pluripotent stem cells could serve as an alternative source for off-the-shelf CAR T cells owing to their unique features of infinite propagation potential and pluripotency. In a 2013 article in *Nature Biotechnology*, Sadelain and colleagues at Memorial Sloan Kettering Cancer Center (100) generated induced pluripotent stem cell (iPSC) clones called T-iPSCs by transducing peripheral blood T cells from a healthy volunteer with two retroviral vectors, each encoding two of the reprogramming factors KLF4, SOX2, OCT-4, and C-MYC. One T-iPSC clone was then stably transduced with CD19-specific CAR and subsequently induced to differentiate into a T lymphoid lineage by using a multistep 30-day culture protocol. While this approach might seem overly complicated at first sight, iPSC-based allogeneic T cell immunotherapy offers unique advantages over manufacturing allogeneic CAR T cells from healthy donors or using autologous T cells: (*a*) Complex genetic cell engineering (genome editing, CAR transduction) needs to be done only once and gives a renewable engineered iPSC line that can be validated and banked, and (*b*) clones can be screened from a pool of cells and only the top-performing clones that meet the standards for overall quality and safety (e.g., no evidence of off-target genomic modification or biallelic disruption of the TCR) can be selected to generate a master engineered iPSC bank for GMP production of the CAR product. Whether this platform can address the commercial scalability challenges of conventional CAR T cell therapy remains to be seen. Fate Therapeutics, one of the developers of iPSC-derived immunotherapies, chose to test their allogeneic NK cell product (FT-516) in patients first (ClinicalTrials.gov identifier NCT04023071), possibly because generating NK cells from iPSCs has proven to be easier than producing antigen-specific cytotoxic T cells (101). Although the first steps of differentiation toward hematopoietic stem and progenitor cells (HSPCs) are similar to those of T differentiation, commitment to the NK lymphoid lineage is less complicated and does not require the presence of Notch signaling. Indeed, generation of T cells from iPSCs is complicated and requires comprehensive understanding of developmental biology and immunology to recapitulate every key event that occurs during T cell commitment. Differentiation of T cells from iPSC-derived HSPCs under feeder- and serum-free conditions has not been reported despite the availability of culture conditions for bone marrow or cord blood HSPCs (102, 103). Therefore, manufacturing protocols need to be established to allow for efficient clinical-scale production of GMP-grade iPSC-derived T cells. Also, the proper phenotype and functional maturity of the generated T cells have to be ensured, as does an antitumor potential comparable with that of natural T cells. Finally, as with all iPSC-derived cellular products, the potential risk of malignant transformation due to contamination with undifferentiated iPSCs has to be minimized, for example, with the use of suicide genes such as the iC9/chemical inducer of dimerization system (104).

## CONCLUSIONS AND PERSPECTIVES

Combining the power of cell therapy with the convenience of a pharmaceutical to create a product that can be brought to a large patient population would be a pivotal achievement in immunotherapy. We envision an off-the-shelf injectable reagent that equips circulating T cells with disease-specific receptors in a minimally disruptive manner, requiring neither chemotherapy preconditioning of the patient nor leukapheresis to harvest lymphocytes as in adoptive cell transfer. Just as for a conventional drug, with this new treatment modality the patient could be easily redosed for as long as medically necessary. The two types of reprogramming reagent highlighted in this review—lymphocyte-targeting nanoparticles and lentiviral vectors—can be manufactured in bulk in a centralized approach. This should substantially lower the treatment costs to levels that allow competition with small molecule pharmaceuticals such as chemotherapy or antiviral compounds, which can cost $50,000 to $80,000 per cycle (105, 106). However, if such T cell reprogramming products are to find their way into routine clinical practice, hurdles associated with stringent regulatory requirements and a complicated intellectual property landscape need to be overcome.

Efficient and selective in situ reprogramming of patient T cells requires highly engineered products containing multiple functional components, such as a lymphocyte-targeting moiety, nucleic acid encoding the disease-specific CAR, and some sort of carrier/condensation matrix, which can be a biopolymer in the case of synthetic nanoparticles or the capsid and envelope for lentiviral vectors. Characterization of the final product containing all components can be daunting, even for a seasoned scientist with expertise in analytical chemistry. At a minimum, characterization should include the following: size (and size distribution), zeta potential, particle concentration, targeting quantification, nucleic acid loading efficiency, coating integrity and quantification, purity (such as residual products from reactions), stability, batch-to-batch consistency, and sterility and endotoxin levels (107). In addition, the US Food and Drug Administration will assess the following factors prior to granting an Investigational New Drug approval for clinical studies (108): (*a*) understanding the mechanism by which the physicochemical properties of the material impact its biological effects (e.g., effect of particle size on pharmacokinetic parameters); (*b*) understanding the in vivo release mechanism based on the material’s physicochemical properties; (*c*) predictability of in vivo release based on established in vitro release methods; (*d*) physical and chemical stability; (*e*) maturity of the nanotechnology (including manufacturing and analytical methods); (*f*) potential impact of manufacturing changes, including in-process controls and the robustness of the control strategy, on critical quality attributes of the drug product; and (*g*) dissolution, bioavailability, distribution, biodegradation, and accumulation data and their predictability based on physicochemical parameters and animal studies.

As alluded to above, the highly competitive (and litigious) intellectual property landscape could further complicate clinical translation of multicomponent nanomedicines. There have been a large number of patent filings with claims to various aspects of nanoparticle delivery of nucleic acid and polymer compositions. Moreover, the CAR T cell patent landscape is becoming increasingly crowded, and this will inevitably lead to narrower claims being granted (if any) in many cases. Ultimately, translating the concept of programming disease-fighting lymphocytes in situ into novel therapeutics for cancer patients will likely require joint efforts involving industry leaders in gene therapy, bioengineering/nanotechnology, and T cell therapy. Ongoing legal wrangles over intellectual property rights covering CAR T cell technology (109) and intellectual disputes over nanoparticle drug-delivery systems raise concerns about whether the biotechnology industry is prepared to merge resources, inventions, and know-how from materials science, immunology, and gene therapy to bring to market alternative cancer treatment strategies. This is unfortunate, because without collaborative invention at the industry level, promising findings that could lead to paradigm-shifting cancer interventions produced by cross-disciplinary research may be stalled at the preclinical stage. Our hope is that the participants in these arenas will recognize the many ways that uniting disciplines and know-how will expedite efforts to develop valuable therapeutics to program antigen-recognizing capabilities into lymphocytes circulating in vivo for the benefit of countless lives.
